# CBCT-Guided Iliosacral Screw Osteosynthesis in a Pregnant Woman: A Case Report and Literature Review

**DOI:** 10.3390/jpm16050235

**Published:** 2026-04-28

**Authors:** Bastien Chalamet, Jean-Baptiste Pialat, Anthony Viste, Didier Defez, Pierre-Adrien Bolze, Nicolas Stacoffe

**Affiliations:** 1 Department of Radiology, Hospices Civils de Lyon, Centre Hospitalier Lyon Sud, 69310 Pierre-Benite, France; jean-baptiste.pialat@chu-lyon.fr (J.-B.P.); nicolas.stacoffe@chu-lyon.fr (N.S.); 2 Faculté de Médecine Lyon-Sud, Université Lyon 1, 69149 Oullins, France; anthony.viste@chu-lyon.fr; 3 Department of Orthopedic Surgery, Traumatology and Sports Medicine, Hospices Civils de Lyon, Centre Hospitalier Lyon Sud, 69310 Pierre-Benite, France; 4 Department of Medical Physics, Hospices Civils de Lyon, Centre Hospitalier Lyon Sud, 69310 Pierre-Benite, France; didier.defez@chu-lyon.fr; 5 Department of Gynecology and Obstetrics, Hospices Civils de Lyon, Centre Hospitalier Lyon Sud, 69310 Pierre-Benite, France; pierre-adrien.bolze@chu-lyon.fr

**Keywords:** CBCT-guided percutaneous fixation, pelvic ring instability, interventional radiology-assisted osteosynthesis, fetal dosimetry, radiation risk management

## Abstract

**Objectives**: Management of unstable pelvic fractures during pregnancy presents a major therapeutic challenge, requiring careful multidisciplinary evaluation to balance maternal benefits and fetal radiation risks. **Methods**: We report the case of a 32-year-old patient who presented with a pelvic fracture due to a road traffic accident at three months of pregnancy. A left sacroiliac osteosynthesis was performed to treat a left sacroiliac diastasis with pelvic osteosynthesis using a trans-iliosacral approach under cone-beam CT (CBCT) guidance using a very-low-dose protocol. Radiation parameters and fetal dose estimates were calculated in advance in collaboration with a medical physicist. Tight beam collimation, a reduced field of view, and minimization of fluoroscopic checks were applied to keep fetal exposure as low as reasonably achievable. This article aims to demonstrate the feasibility of managing a complex pelvic fracture using interventional radiology and to review the literature on management options and gestational age-dependent fetal risks. **Results**: The estimated cumulative fetal dose from initial imaging, open surgery, and CBCT-guided osteosynthesis remained below 70 mGy using a pregnant phantom (Duke Organ Dose–Dosewatch–General Electric system), which is below thresholds associated with deterministic effects. The procedure achieved optimal screw positioning with less than 40 s of fluoroscopy. Maternal postoperative recovery was favorable, and follow-up revealed normal fetal development. **Conclusions**: This case demonstrates that CBCT-guided percutaneous iliosacral screw fixation can be safely performed during pregnancy with meticulous planning, dose-reduction strategies, and multidisciplinary collaboration, maintaining fetal radiation exposure below accepted safety thresholds.

## 1. Introduction

Management of unstable pelvic fractures during pregnancy represents a major therapeutic challenge and requires careful multidisciplinary assessment to balance maternal benefit against potential fetal radiation exposure [1,2]. This issue is particularly critical when pelvic osteosynthesis is required, as the fetus may lie directly within or near the irradiation field [3].

Pelvic ring and acetabular fractures are severe injuries associated with substantial morbidity and mortality, with potentially major consequences for long-term functional outcomes. Pelvic ring fractures may result in gait instability and chronic pelvic pain, whereas acetabular fractures are associated with early hip arthropathy, acetabular protrusion, or hip instability, particularly when the posterior column or wall is involved [4].

Osteosynthesis is a widely used surgical technique for fracture fixation. In recent years, the development of minimally invasive and percutaneous approaches has expanded treatment options for pelvic stabilization. These techniques increasingly rely on advanced intraoperative imaging modalities, such as cone-beam computed tomography (CBCT), to enhance accuracy while reducing complication rates [5].

In pregnant patients, the indication for osteosynthesis must be carefully weighed because of potential fetal exposure to ionizing radiation. According to the recommendations of the International Commission on Radiological Protection (ICRP), in utero radiation exposure may lead to deterministic effects above established dose thresholds—such as pregnancy loss, congenital malformations, or neurodevelopmental impairment—or to stochastic effects without a threshold, mainly carcinogenesis, with risks depending on absorbed dose and gestational age [6].

Although unstable pelvic fractures during pregnancy are rare and typically result from high-energy trauma, conservative management is not without limitations. While it avoids anesthesia and radiation exposure, prolonged immobilization may lead to significant maternal complications, including deconditioning, thromboembolic events, and long-term pelvic deformity, which may compromise functional recovery [7].

In this context, we report a case illustrating the feasibility of managing a complex unstable pelvic fracture in a pregnant woman while maintaining fetal radiation exposure below the recommended threshold of 100 mGy, followed by a review of the literature [3]. This case was considered relevant as it highlights the potential benefits of surgical stabilization, even in highly complex clinical scenarios.

Despite the specific concerns related to radiation exposure during pregnancy, a multidisciplinary discussion involving orthopedic surgeons, radiologists, obstetricians, and a medical physicist enabled the development of a personalized treatment strategy. This collaborative approach ensured optimal mechanical stabilization while prioritizing maternal and fetal safety. The present case demonstrates that, with meticulous planning and tailored dose-reduction strategies, effective pelvic fracture fixation during pregnancy is both feasible and beneficial for long-term functional outcomes.

## 2. Case Report

**Figure 1:**
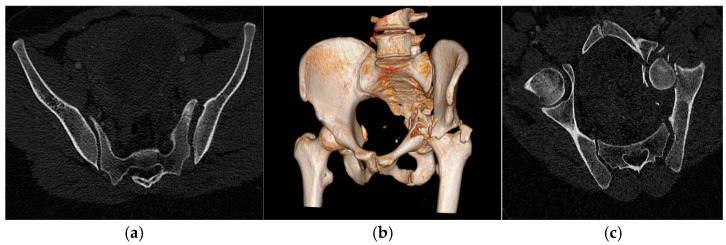
(**a**). Axial view showing disjunction of the left sacroiliac joint (open-book fracture). (**b**). 3D reconstruction providing an overview of the pelvic trauma. (**c**) Oblique multiplanar axial reconstruction showing disruption of the pelvic ring caused by a comminuted fracture of the left acetabulum.

A 32-year-old woman was admitted to the emergency department after being struck by a car. She was in the early second trimester of pregnancy, and the initial fetal ultrasound demonstrated normal viability with no evidence of traumatic complications. On arrival, the patient presented with severe left pelvic pain and marked shortening of the left lower limb. Imaging revealed a complex injury pattern involving the left hemi-pelvis. She had a displaced acetabular fracture with protrusion of the femoral head into the pelvic cavity, associated with a disjunction of the left sacroiliac joint. This corresponded to a Tile B [8] pelvic ring injury, unstable in the horizontal plane (Figure 1).

Clinically, the patient reported intense pain exacerbated by the slightest movement. Her medical history was unremarkable apart from mild asthma, carpal tunnel syndrome, and a past oocyte retrieval as part of assisted reproductive treatment. The coexistence of high-energy pelvic trauma and pregnancy required careful multidisciplinary coordination to ensure both maternal safety and fetal protection. Given the mechanical instability and risk of progressive displacement, prompt but meticulously planned management was deemed essential to optimize functional outcomes while minimizing radiation exposure.

After interdisciplinary consultation with the orthopedic surgeon and gynecologists, it was decided to proceed first with open surgery to reduce the left acetabular fracture through a posterior approach using a plate. Subsequently, a left iliosacral osteosynthesis was planned in interventional radiology to stabilize the posterior arch. The two interventions were carried out between the second and fifth day after the injury. The goal was to enable the patient to sit up in bed and regain full weight-bearing capacity after three months.

A CT body scan and radiographs performed during the initial management of the patient led to a fetal irradiation dose estimated under 50 mGy ± 5 mGy using a pregnant phantom (Duke Organ Dose–Dosewatch–General Electric system, DoseWatch 3.1). The maximum allowable exposure for a fetus should not exceed 200 mGy to avoid an increased risk of intellectual disability, with the range of uncertainty between 100 and 200 mGy. Before the procedures, the patient was informed of the risk of fetal radiation exposure in obstetric and interventional radiology consultations. Between each intervention, a fetal viability ultrasound was performed to ensure that the fetus remained in good condition. Oral and written consent was obtained from her in advance of all procedures.

The osteosynthetic fixation was performed under general anesthesia in an angiography room, including a CBCT guidance mode (Azurion Flex, PHILIPS, Koninklijke Philips N.V., Amsterdam, The Netherlands). The acquisition protocol was adapted, using a low-dose protocol (180° rotation, 125 kV, and 430 mA) and a small field of exposition to achieve the lowest reasonably achievable dose (ALARA) for a personalized treatment, which led to sufficient image quality and minimized fetal exposure. The patient was positioned in a prone position using foam wedges to limit pressure on the fetus and to allow correct access to the left sacroiliac joint to optimally position the fixation screw.

**Figure 2:**
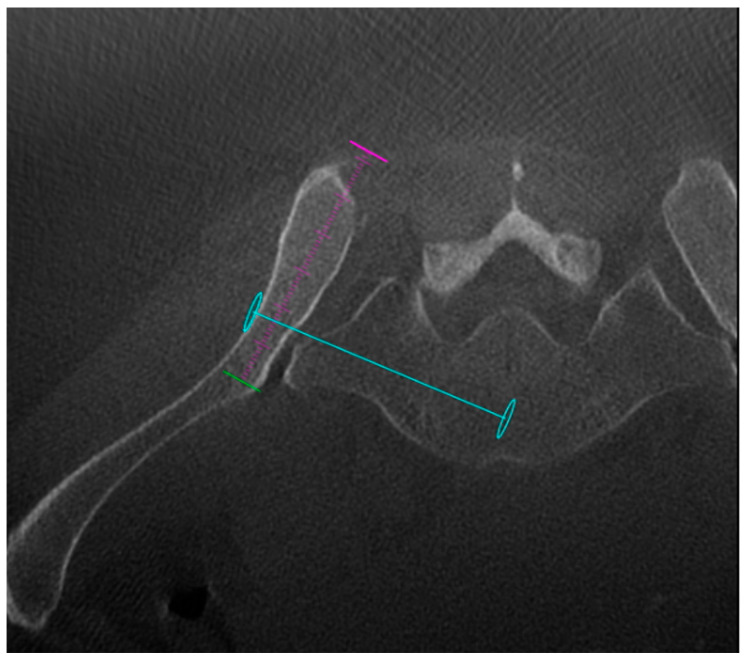
Initial CBCT images. Axial view shows the trajectory chosen by the operator, visible as a blue line, with the entry point at the iliac wing (the most lateral blue circle) and the distal point at the level of the S1 promontory (central blue circle). The purple and green circle indicates the projection line of the posterior iliac wing.

An initial CBCT acquisition was performed to assess anatomical variations and to plan the optimal screw trajectory (Figure 2).

**Figure 3:**
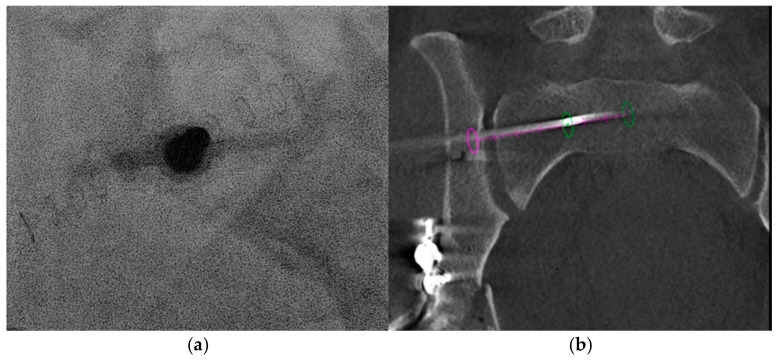
Fluoroscopy and CBCT guidance during the procedure. (**a**). Fluoroscopic guidance showing trocar positioning at a bull’s eye view at the level of the first sacral segment (S1). (**b**). Coronal view with CBCT guidance. The pin is visible in place, with its distal end reaching the promontory. The purple circle indicates the iliac entry point, while the green circle marks the target at the sacral promontory. The display represents the progression view.

A three-dimensional planning and guidance software (XperGuide^®^ R 1.5.5) was then used. The software generated a “bull’s eye” view by aligning the skin entry point with the distal target point (planned final screw tip), and a progressive view that allowed real-time monitoring of screw advancement to the target depth. A breakaway Shiba needle (19 G) (Strim Health care, Margencel, France) was advanced to bone contact in the “bull’s eye” view. After removal of the needle head, a bone trocar (TOCR, 8 G, Strim Health care, Margencel, France) was inserted over the needle (Figure 3a). Advancement through the bone was performed with gentle hammering. We were able to confirm the correct positioning of the trocar using simple fluoroscopic control. A 3.2 mm diameter, 300 mm long guidewire was then positioned through the trocar. A second CBCT (Figure 3b) was performed to confirm the correct positioning of the trocar and to calculate the screw’s length (70 mm).

**Figure 4:**
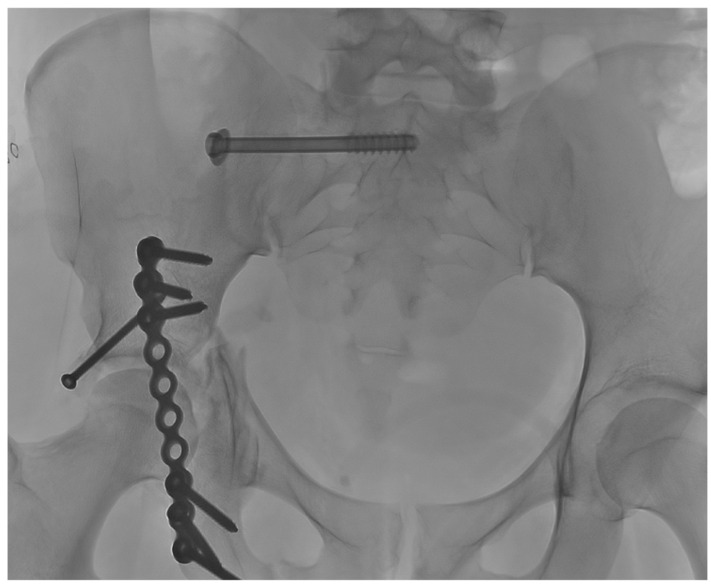
Postoperative X-ray shows the correct positioning of the screw.

A titanium cannulated screw (Stryker, ASNIS III, Kalamazoo, MI, USA) was inserted over the guidewire and locked into the cortical bone. We used partially threaded 8 mm screws with a metal washer to apply some compression and reduce sacroiliac joint diastasis. At the end of the procedure, the antero-posterior view confirmed optimal positioning of the fixation screw (Figure 4).

In total, the time of fluoroscopy was less than forty seconds. The pre- and post-procedure fetal viability ultrasound, carried out by our obstetrics team, showed no abnormalities. During the procedure, we deliberately minimized the use of fluoroscopy in order to limit fetal radiation exposure as much as possible. Every imaging step was carefully planned in advance, and the beam was collimated tightly to keep the fetus outside the irradiation field whenever feasible. Protecting the fetus remained a constant priority throughout the intervention, guiding each technical decision and ensuring that only essential fluoroscopic acquisitions were performed.

CBCT acquisition led to a total Dose Area Product (DAP) of 0.027742 Gy·cm^2^. Using Monte Carlo simulations on a model of a non-pregnant woman (PCXMC 2.0.1, STUK 2008), the maximum dose estimated for the uterus was 20 mGy.

This dose is around three times lower than the “standard” radiation dose for this type of operation (with an average of 83.8 mGy for patients with the same characteristics operated on in the same room by the same team) due to maximal optimization of all parameters.

The patient was also assessed using a body CT scanner during early management after the car accident (dose of 44 mGy), and open surgery (anterior osteosynthesis) in the operating room using fluoroscopy guidance (<5 mGy).

In total, the estimated dose delivered to the fetus was less than 70 mGy, which remains within the safety zone concerning the risk of malformation (Table 1).

We collectively decided to perform the procedure after a preliminary calculation of the estimated radiation dose, taking into account the irradiation already received during previous procedures. The patient’s follow-up did not reveal any complications of the open surgery or percutaneous procedure. After 2 months, the follow-up radiograph did not show any displacement or signs of consolidation of the pelvic and acetabular bone. A prophylactic cesarean section was decided by the multidisciplinary staff rather than a vaginal delivery due to pelvic ring narrowing after residual impaction of the acetabular bone and because of the fixation of one of the sacroiliac joints. She could give birth by caesarean section in good condition and without any problems for her baby.

The patient was evaluated at 3 and 7 months postoperatively. At 3 months, she was ambulating with crutches and reported complete resolution of pain. At 7 months postoperatively, corresponding to 3 months postpartum, she had regained full independence in daily activities and ambulation without assistance. Her infant, currently 4 months old, is in good health with no abnormalities detected during follow-up. Ethical approval is not required due to the French Jardé Law (Code de la santé publique), which specifies that a single case report is generally not considered research involving human subjects.

## 3. Discussion

Pelvic fractures resulting from trauma during pregnancy are uncommon but require early and thorough evaluation because of the potentially severe consequences for both the mother and the fetus [9]. Reported mortality rates reach approximately 9% for the mother and up to 35% for the fetus, highlighting the seriousness of these injuries [10].

This case is noteworthy as it demonstrates the feasibility of performing complex pelvic osteosynthesis during pregnancy. It underscores the importance of close collaboration among treating physicians to tailor management to the individual patient. In rare and atypical situations, strict adherence to standard protocols may be insufficient, making a personalized approach essential. Multidisciplinary discussions facilitate careful risk assessment, consideration of alternative therapeutic strategies, and informed decision-making based on patient-specific factors. Such an approach enhances treatment efficacy while ensuring safe, patient-centered care in complex clinical scenarios.

In pregnant women, pelvic fractures are most commonly stable injuries (Tile A). Unstable fractures are typically associated with high-energy trauma, most often related to road traffic accidents. Sports-related pelvic fractures are less frequent, as high-risk activities are generally avoided during pregnancy. Metabolic disorders and pregnancy-related hormonal changes may predispose a patient to insufficiency fractures; however, these rarely result in significantly displaced fractures.

Fractures during pregnancy are preferentially managed conservatively in order to avoid fetal exposure to anesthesia and ionizing radiation. Radiation exposure, whether in a medical or occupational setting, remains a major source of concern for expectant mothers. Nevertheless, in selected cases—such as complex unstable pelvic fractures—the risk–benefit balance of surgical osteosynthesis must be carefully considered.

The introduction of percutaneous, fluoroscopy-guided techniques has represented a major advance in pelvic fracture management. However, conventional two-dimensional imaging has inherent limitations. In this context, cone-beam computed tomography (CBCT) offers high-precision three-dimensional reconstructions and “bull’s eye” trajectory views, allowing real-time visualization of screw placement while helping to optimize accuracy and minimize radiation exposure.

Currently, the literature on pelvic osteosynthesis performed under interventional radiology guidance using CT or CBCT remains limited. Only a small number of articles and case series describe surgical pelvic osteosynthesis in pregnant patients [11,12,13]. Therefore, the present case adds valuable data by demonstrating that effective stabilization can be achieved without exceeding recommended fetal radiation dose thresholds.

In our case, limited pelvic stabilization was considered necessary to avoid prolonged bed rest and prevent long-term pelvic deformity, which could lead to persistent pain and functional impairment. Ionizing radiation is associated with two types of biological effects: deterministic and stochastic. Deterministic effects are dose-dependent and have a threshold, with teratogenic risks varying according to gestational age and absorbed fetal dose. During the very early stage after conception, the “all-or-nothing” principle applies, whereby exposure either results in pregnancy loss or normal development; this principle is limited to the first week following conception. When fetal exposure remains below 100 mGy, the risk is considered equivalent to that of a pregnancy without exposure.

For doses exceeding 200 mGy, the risk of congenital malformations increases during the period of organogenesis, between the 1st and 8th weeks of gestation. Between 8 and 16 weeks, the central nervous system is particularly sensitive, with a risk of neurodevelopmental impairment, including a potential reduction in intellectual quotient, which gradually decreases until birth. In such situations, medical termination of pregnancy may be discussed with the parents.

Stochastic effects, primarily carcinogenesis and childhood leukemia, have no defined threshold and may occur even at low radiation doses, with risk increasing proportionally to the dose received. Consequently, an increased cancer risk cannot be completely excluded even below 100 mGy [6,14].

**Figure 5:**
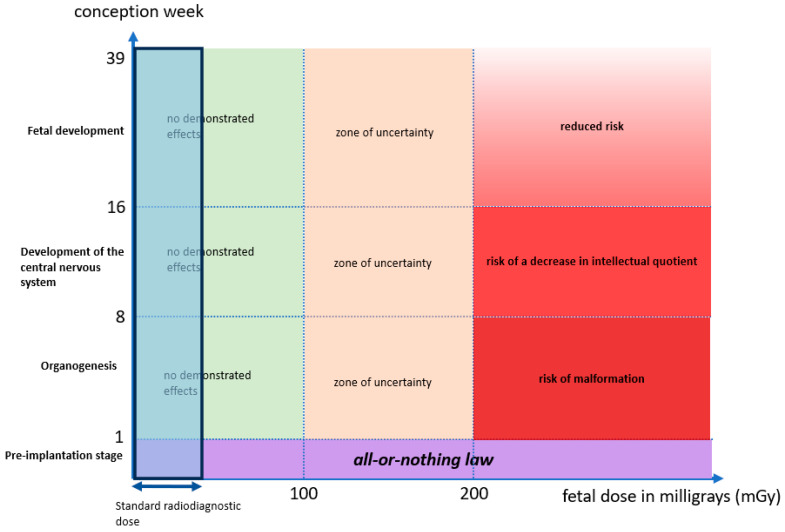
Graph summarizing the risk of malformations based on the week of conception and the dose received by the fetus.

A graph is presented summarizing the risk of malformations based on the week of conception and the dose received by the fetus (Figure 5).

Typical radiation doses associated with diagnostic medical imaging are generally below 100 mGy, providing a substantial safety margin with respect to deterministic fetal effects. However, interventional procedures involving ionizing radiation, particularly those performed under CT or CBCT guidance, raise legitimate concerns regarding the potential for higher cumulative doses, especially when standard acquisition parameters are used. Nevertheless, by applying optimized low-dose protocols that maintain sufficient image quality and by continuously monitoring fetal exposure, radiation doses can be kept within a range considered acceptable.

Such an approach allows optimal management of complex pelvic fractures, which may otherwise result in significant long-term functional impairment if inadequately treated. These therapeutic decisions must be made through collegial, multidisciplinary discussion and accompanied by clear and comprehensive information provided to the patient. Importantly, this strategy also opens new perspectives for the management of similar fractures in pregnant patients.

The present case demonstrates that comprehensive and appropriate treatment of unstable pelvic fractures during pregnancy is feasible without exposing either the mother or the fetus to excessive risk, provided that meticulous planning and strict optimization of radiation dose are applied.

## 4. Conclusions

This case demonstrates that CBCT-guided percutaneous iliosacral screw fixation can be safely performed during pregnancy when supported by meticulous planning and rigorous dose-reduction strategies. By optimizing acquisition parameters, minimizing fluoroscopic use, and involving a multidisciplinary team—including interventional radiology, orthopedic surgery, obstetrics, and medical physics—it is possible to maintain fetal radiation exposure well below accepted safety thresholds while achieving accurate and stable fracture fixation.

CBCT guidance offers a valuable alternative to conventional fluoroscopy or open surgery in complex pelvic injuries, providing precise three-dimensional trajectory planning and real-time assessment of implant positioning. Integrated dose-reduction systems may play an important future role in optimizing radiation exposure during interventional procedures.

Although evidence remains limited, this case highlights the potential of advanced percutaneous techniques to improve maternal outcomes without compromising fetal safety. Further clinical experience and larger case series are needed to refine guidelines and better define the place of CBCT-guided osteosynthesis in the management of pelvic fractures during pregnancy.

## Figures and Tables

**Table 1:** Total estimated dose.

Examination/Procedure	Context	Type of Exposure	Estimated Fetal Dose
Body CT scan	Early management after car accident	Whole-body CT	44 mGy
Open surgery (anterior osteosynthesis)	Operating room	Intraoperative fluoroscopy	<5 mGy
Percutaneous osteosynthesis	Interventional radiology	Fluoroscopy + CBCT	≈20 mGy
Total estimated fetal dose	Cumulative exposure	-	<70 mGy

## Data Availability

The original contributions presented in this study are included in the article. Further inquiries can be directed to the corresponding author.

## Abbreviations

The following abbreviations are used in this manuscript:

CBCT	CONE BEAM CT
